# Continuous-flow enantioselective α-aminoxylation of aldehydes catalyzed by a polystyrene-immobilized hydroxyproline

**DOI:** 10.3762/bjoc.7.172

**Published:** 2011-10-31

**Authors:** Xacobe C Cambeiro, Rafael Martín-Rapún, Pedro O Miranda, Sonia Sayalero, Esther Alza, Patricia Llanes, Miquel A Pericàs

**Affiliations:** 1 Institute of Chemical Research of Catalonia (ICIQ). Avda. Països Catalans, 16. E-43007, Tarragona, Spain; 2 Departament de Química Orgànica, Universitat de Barcelona. Avda. Martí I Franqués, 1. E-08028, Barcelona, Spain

**Keywords:** α-aminoxylation, continuous flow, packed-bed reactors, polystyrene-immobilized catalysts, proline

## Abstract

The application of polystyrene-immobilized proline-based catalysts in packed-bed reactors for the continuous-flow, direct, enantioselective α-aminoxylation of aldehydes is described. The system allows the easy preparation of a series of β-aminoxy alcohols (after a reductive workup) with excellent optical purity and with an effective catalyst loading of ca. 2.5% (four-fold reduction compared to the batch process) working at residence times of ca*.* 5 min.

## Introduction

Optically active α-hydroxycarbonyl moieties are highly versatile functional synthons and are present in a wide range of biologically active natural products [1–2]. Traditional strategies for the preparation of these kinds of synthons involves the oxidation of preformed enolates, both with the use of chiral auxiliaries (chiral, enantiopure enolates and achiral oxidizing agents, or achiral enolates and chiral, enantiopure oxidizing agents) [3–5] and by Mukaiyama-type catalytic processes involving preformed achiral enolate equivalents and achiral oxidants, with chiral enantiopure Lewis acids as catalysts [6–11].

Worth noting is the contribution made by Yamamoto et al., who introduced the use of nitrosobenzene as an electrophilic source of oxygen in the aminoxylation of preformed tin enolates catalyzed by a chiral, silver-based Lewis acid [12]. With this methodology an α-aminoxyketone was obtained that could be readily transformed into the corresponding α-hydroxyketone in the presence of catalytic amounts of CuSO_4_·5H_2_O in methanol.

**Scheme 1:**
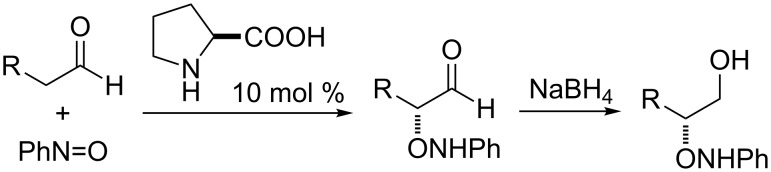
Proline-catalyzed direct enantioselective α-aminoxylation of aldehydes.

This strategy was soon extrapolated to the field of organocatalysis, leading in 2003 [13] to the first organocatalytic approaches to the direct enantioselective α-aminoxylation of carbonyl compounds and, shortly after, to the implementation of the α-aminoxylation of aldehydes with proline as catalyst [14–15] (Scheme 1). In 2004, the scope of the reaction was extended to ketones [16–17] and the reaction was subsequently applied to the synthesis of several biologically active compounds [18–20].

Proline is the most frequently used catalyst in α-aminoxylation reactions, but other catalytic species have also been developed and used. Chiral secondary amines, such as substituted pyrrolidines other than proline [21], binaphthyl-derived amines [22–24], pyrrolidin-2-yl-tetrazoles [25–28], thiaproline [29], 2-aminomethylpyrrolidine sulfonamide [30] and sulfonylcarboxamide [31], have been successfully used to promote the reaction. As a matter of fact, the rapid development of the methodology for the asymmetric organocatalyzed α-aminoxylation of aldehydes and ketones experienced in the last few years has transformed it into a powerful, reliable and environmentally friendly method for the synthesis of α-hydroxyaldehydes and ketones [32–34].

**Figure 1:**
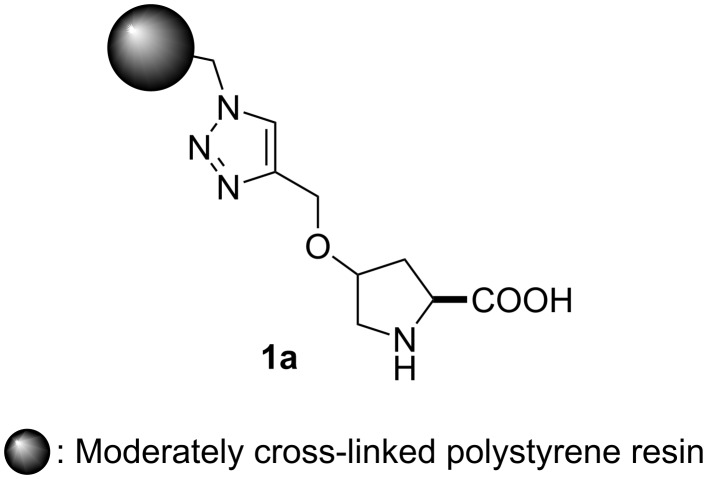
Polystyrene-immobilized hydroxyproline **1a**.

Despite its extreme simplicity and very high enantiocontrol, organocatalysis has frequently been the subject of criticism for the relatively low catalytic activities, which hence require the use of catalyst loadings as high as 30 mol % [35–36] for the achievement of high conversions in reasonable reaction times. A very reliable and convenient strategy to overcome this limitation is the development of reusable immobilized catalysts, thus allowing important reductions of the “effective” catalyst loading (through recycling and repeated use). Different approaches have been used for the development of immobilized analogs of proline and other organic catalysts. Among them, a prominent position is occupied by catalysts covalently immobilized onto insoluble, cross-linked polymers [37–40]. An interesting development arising from this strategy is the polystyrene-immobilized 4-hydroxyproline **1a** (Figure 1), reported by our group as an extremely efficient and reusable catalyst for the direct enantioselective aldol [41] and Mannich reactions [42] as well as for the α-aminoxylation of carbonyl compounds under batch conditions [43]. Interestingly, the triazole linker between the polymer and the active unit, inherently resulting from the Cu(I)-catalyzed cycloaddition of azides and alkynes (CuAAC) used as the immobilization strategy [44–46], led to improved efficiency, both in terms of catalytic activity and asymmetric induction, and different behaviour of the resulting materials in terms of hydrophilicity or hydrophobicity [47–50].

An even better alternative for improving the productivity of catalytic species results from the implementation of heterogenized catalysts in continuous-flow reactors. Flow chemistry has experienced a very important development in the last ten years as an emerging technology for organic synthesis [51–66]. It offers as its main advantages facile automation and excellent heat and mass transfer, rendering the scale-up of a process a trivial task, in contrast with the obstacles always met in the scale-up of batch processes [67–71].

The combination of flow chemistry with solid-supported catalysts allows the advantages inherent to both technologies to be added together. Thus, the physical immobilization of the catalyst in a packed-bed reactor allows it to be submitted constantly to the reaction conditions, avoiding possible degradation of the catalyst during operations other than the reaction itself (washing, drying, storage, etc.) [72]. This frequently leads to a significant extension of the catalyst’s lifetime. In addition, any further processing for the separation of the catalyst from the reaction mixture is no longer needed. The combined effect of these factors is an improvement in the catalyst productivity, with a corresponding reduction in the cost of any given process [42,50,73–75].

Herein we present the implementation of a continuous-flow packed-bed reactor with heterogenized catalyst **1** for the fast, enantioselective, direct α-aminoxylation of aldehydes.

## Results and Discussion

**Scheme 2:**
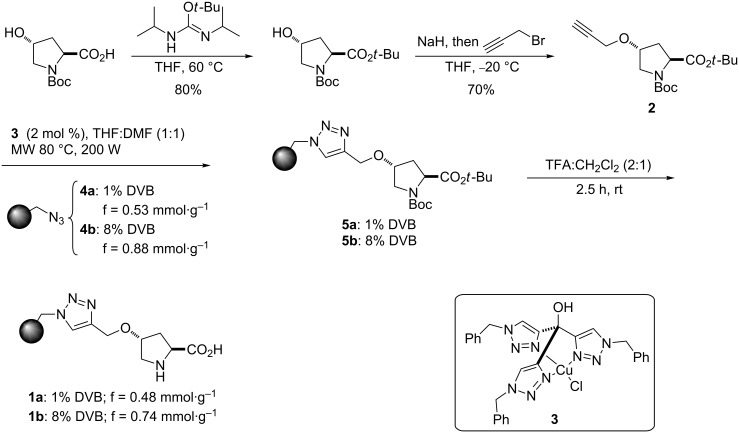
Preparation of the immobilized catalysts **1a** and **1b**.

The preparation of the immobilized catalysts **1a** and **1b** was easily achieved by a modification of the reported procedure [41–43], with the tris(triazolyl)methyl copper complex **3** [76] as the catalyst for the CuAAC reaction between azidomethylpolystyrene, prepared from a Merrifield resin, and propargyloxyproline derivative **2**, which was readily obtained in two steps from commercially available (2*S*,4*R*)-*N*-Boc-4-hydroxyproline (Scheme 2).

Based on previous experience, we considered that variations in the degree of cross-linking of the support resins could have an important effect on their mechanical stability [66], this resulting in differences in their performance. In order to assess this issue, we prepared two different immobilized catalysts, starting in one case (**1a**) from commercially available Merrifield resin containing 1% of 1,4,-divinylbenzene (DVB) as a cross-linking agent and in the other case (**1b**) from a home-made Merrifield resin with 8% DVB (prepared by radical copolymerization of styrene, 4-chloromethylstyrene and DVB, under previously reported conditions [77–79]).

It is well known that slightly cross-linked (1–2% DVB) polystyrene is microporous in nature and readily swells in a variety of solvents, leading to gel formation. For catalytic resins, this ensures contact of the reactants in solution with essentially all the catalytic sites and, accordingly, high activity. This positive characteristic, however, can be countered by a poor mechanical stability that can lead to structural collapse and deactivation under the pressure applied for flow operation. Polystyrene cross-linked with 8% DVB still shows significant swelling with a variety of solvents, such that its behaviour can be considered as being intermediate between those of microporous and macroporous resins. We accordingly expected that the catalyst immobilized on such a more heavily cross-linked resin, such as **1b**, would retain an important level of activity while having less of a tendency for structural collapse under the flow conditions, and that, as a consequence, the useful lifetime of the catalyst would be significantly improved.

After final deprotection, immobilized catalysts **1a** and **1b** were obtained with functionalizations (f) of 0.48 and 0.74 mmol·g^−1^, respectively.

**Scheme 3:**
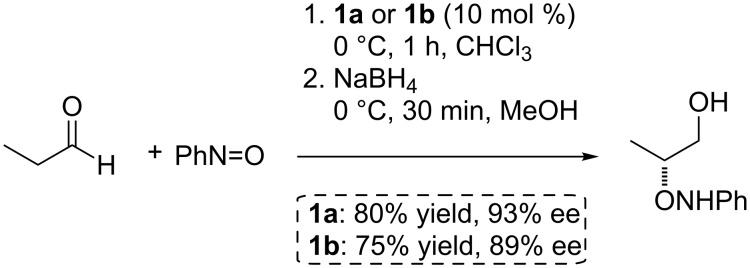
Direct enantioselective α-aminoxylation of propanal catalyzed by resins **1a** and **1b**.

Both resins were evaluated as catalysts for the α-aminoxylation reaction of aldehydes with nitrosobenzene, with propanal as a benchmark substrate, under standard batch conditions [43]. In both cases the product, isolated in the form of its reduced β-aminoxy alcohol due to the intrinsic instability of α-aminoxy aldehydes, was obtained with good yields and enantioselectivities in short reaction times (Scheme 3). Although catalyst **1a** afforded better results both in terms of enantioselectivity and catalytic activity, we were pleased to observe that the more heavily cross-linked catalytic resin **1b** exhibited a catalytic activity almost identical to that of **1a**, with only a marginal decrease in enantioselectivity.

With these results in hand, we set up flow conditions for the two catalysts to study the scope and limitations of their use in the α-aminoxylation of aldehydes.

**Figure 2:**
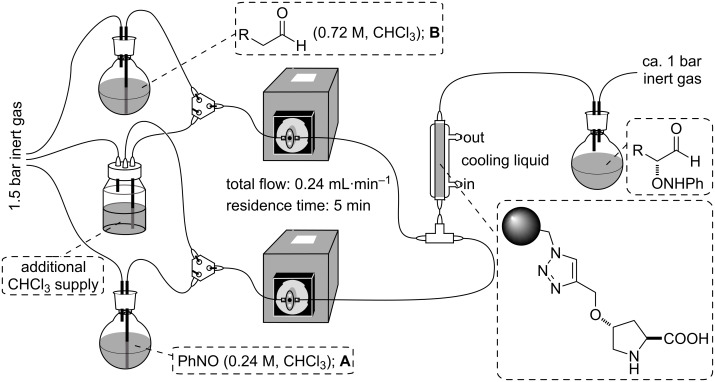
Experimental setup for the continuous-flow α-aminoxylation of aldehydes.

For the continuous-flow experiments, following our previous experience with similar systems, the instrumental setup shown in Figure 2 was used. The packed-bed reactor consisted of a vertically mounted, fritted and jacketed low-pressure Omnifit glass chromatography column (10 mm pore size and up to a maximal 70 mm of adjustable bed height) filled with 300 mg of swollen resin (ca. 17 mm bed height). Two separate piston pumps were connected to the reactor inlet through a T-shape connector placed right before the reactor, and which acted as a mixing chamber, and a collection flask was connected to the reactor outlet.

During operation, each of the pumps was connected to one of the feeding solutions **A** and **B**; solution **A** containing nitrosobenzene (0.24 M) and 1,1,2,2-tetrachloroethane (0.06 M, internal standard), and solution **B** containing propanal (0.72 M) in chloroform. Both solutions were pumped through the system at a rate of 0.12 mL·min^−1^ each.

Additionally, a supply of pure chloroform was connected to both pumps, for nonreaction operations such as swelling the resin and washing the tubing before and after the reaction. Isothermal operation was ensured by circulation of a cooling fluid at the desired operation temperature (0 °C) through the column jacket. Finally, the collection flask was set in a cold bath at −78 °C, in order to avoid degradation of the aminoxylated products after collection.

The conversion at any given moment was determined from the ^1^H NMR spectra of samples periodically collected from the reactor output. At the end of the experiment the product was reduced with sodium borohydride.

After optimization of the parameters for the continuous-flow process, both catalysts **1a** and **1b** were tested with different substrates, with the results as shown in Table 1.

**Table 1:** Continuous-flow, direct, enantioselective α-aminoxylation of various aldehydes with immobilized catalysts **1a** and **1b**.^a^

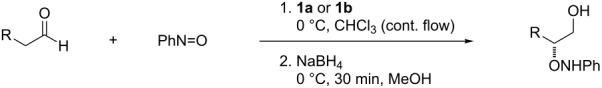								

			Catalyst **1a**			Catalyst **1b**		
Entry	Aldehyde	Time (h)	Conv^b^ (%)	ee^c^ (%)	Productivity^d^	Conv^b^ (%)	ee^c^ (%)	Productivity^d^

1	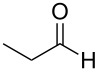	1	75	95	27.3	94	96	16.1
		2	69			90		
		3	67			88		
		4	65			93		
		5	60			82		

2	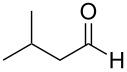	1	83	94	12.6	57	98	9.3
		2	70			63		
		3	76			54		
		4	82			70		
		5	65			49		

3	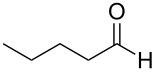	1	49	95	31.4	47	95	3.7
		2	57			46		
		3	43			43		
		4	48			72		
		5	49			–		

4	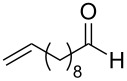	1	68	96	26.7	67	95	9.8
		2	70			39		
		3	63			42		
		4	55			36		
		5	54			43		

5	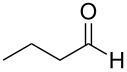	1	95	96	32.7	–	–	–
		2	70			–		
		3	67			–		
		4	69			–		
		5	67			–		

^a^Reactions were performed with 300 mg of resin (0.144 mmol for **1a** or 0.222 mmol for **1b**), 0.24 mL min^−1^ total flow rate (ca. 5 min residence time), 0.12 M concentration of nitrosobenzene and 0.36 M of the corresponding aldehyde. ^b^Instant conversion, determined by ^1^H NMR of samples without any workup. ^c^Determined by HPLC analysis of the reduced (NaBH_4_) product. ^d^In mmol of pure isolated product per mmol of catalyst (accumulated throughout the process).

Both immobilized catalysts **1a** and **1b** provided equally excellent enantioselectivities, which remained completely stable throughout the reaction. On the other hand, regarding catalytic activity, resin **1a**, containing only 1% of the cross-linking agent DVB, exhibited consistently better productivities with the different substrates. It is also worth noting that in the case of catalyst **1a** the conversions registered for the different substrates were not dependent on the chain length (Table 1, entries 3–5) or on the presence of branching in the β-position of the aldehyde (Table 1, entry 2). In the case of catalyst **1b**, lower catalytic activity was observed in all cases (note that the catalyst loading was 0.222 mmol for **1b** while it was 0.144 mmol for **1a**) and, intriguingly, it was strongly dependent on the chain length and branching. Propanal (Table 1, entry 1) gave place to a much higher conversion with resin **1b** than longer-chain (Table 1, entries 3–5) or branched (Table 1, entry 2) aldehydes did. A possible explanation for this behaviour could be the increased rigidity of the resin **1b**, which contains 8% of DVB, causing stable channels and pores of a rather small diameter to be present (macroporous behaviour), instead of the more geometrically tolerant gel in the case of the resin **1a** with 1% of DVB.

Finally, both immobilized catalysts showed only a moderate stability, a slow decrease in the conversion being observed with time in both cases. Contrary to our expectations based on the different degrees of cross-linking, no significant difference in stability was detected between resin **1a** and resin **1b** (the one with 8% DVB being only slightly more stable, as indicated by the conversion versus time data for propanal; see Table 1, entry 1). Although not conclusive, these data suggest that the deactivation process is of a chemical nature, possibly due to the formation of an oxazolidinone between proline and the aldehyde [80–82], rather than a physical denaturation of the polymer. With the most efficient resin (**1a**), ca. 30 mmol of product was isolated per mmol of resin for every flow experiment (5 h), thus meaning a four-fold improvement with respect to the batch process.

## Conclusion

In summary, a packed-bed continuous-flow reactor was designed and implemented, based on polystyrene-immobilized 4-hydroxyproline, for the direct enantioselective α-aminoxylation of simple aldehydes. The system allowed for the first time the medium-scale preparation of a series of α-oxy-substituted aldehydes through a simple flow process involving short residence times. A lack of stability of the catalyst under the employed reaction conditions led to slow deactivation (15–20% in a five-hour reaction cycle), such that further work towards a practical solution of this problem is still required. Research aimed at understanding the principles determining the stability of immobilized catalysts in flow processes and, consequently, at the development of immobilized species with extended life cycle for α-aminoxylation and for other synthetically important reactions is currently underway in our laboratories.

## Experimental

### General procedure for the α-aminoxylation of aldehydes under continuous-flow conditions

Typical conditions for ca. five hours operation with pentanal: A solution (**A**) containing nitrosobenzene (1.29 g, 12 mmol) and 1,1,2,2-tetrachloroethane (317 µL, internal standard) in chloroform (50 mL, 0.24 M in nitrosobenzene) and another one (**B**) containing pentanal (3.8 mL, 36 mmol) in chloroform (50 mL, 0.72 M) were prepared. Both solutions were separately pumped at a rate of 0.12 mL·min^–1^ each through a glass column containing the immobilized catalyst **1a** (300 mg, 0.144 mmol), which had been previously swollen with chloroform. The effluent of the column was collected in a closed flask cooled down to –78 °C and the reaction was monitored by ^1^H NMR of samples taken directly from the effluent.

After the feeding solutions were finished, chloroform was pumped at the same rate throughout the system for 1 h in order to rinse all the remaining materials. The product solution was diluted with 50 mL of methanol and, after the mixture was allowed to warm up to 0 °C, sodium borohydride (1.36 g, 36 mmol) was added in portions. The resulting slurry was vigorously stirred at 0 °C for 30 min and then water (100 mL) was added. The two layers were separated and the organic one was washed with brine (3 × 50 mL), dried with anhydrous sodium sulfate, filtered and concentrated under reduced pressure. The yellow oil obtained was purified by flash chromatography through silica gel with hexane-ethyl acetate mixtures to yield, after removal of the solvents, the β-aminoxy alcohol product as a colourless oil (910 mg, 96% ee).

## Supporting Information

File 1 Detailed experimental procedures for the preparation of the catalysts and chromatographic methods for the determination of the enantiomeric excess of the products.
