# Supplementary material for: Continuous-flow enantioselective α-aminoxylation of aldehydes catalyzed by a polystyrene-immobilized hydroxyproline
Source: Beilstein J Org Chem. 2011 Oct 31;7:1486–93. doi: 10.3762/bjoc.7.172 (PMC3252847; doi:10.3762/bjoc.7.172)
Supplement: File 1 — Detailed experimental procedures for the preparation of the catalysts and chromatographic methods for the determination of the enantiomeric excess of the products. [file Beilstein_J_Org_Chem-07-1486-s001.pdf]

Supporting Information

for

Continuous-flow enantioselective α-aminoxylation of aldehydes catalyzed by a polystyrene-immobilized hydroxyproline

Xacobe C. Cambeiro[1], Rafael Martín-Rapún[1], Pedro O. Miranda[1], Sonia Sayalero[1], Esther Alza[1], Patricia Llanes[1], Miquel A. Pericàs*[,1,2]

Address: [1]Institute of Chemical Research of Catalonia (ICIQ). Avda. Països Catalans, 16. E-43007, Tarragona, Spain and [2]Departament de Química Orgànica, Universitat de Barcelona. Avda. Martí I Franqués, 1. E-08028, Barcelona, Spain

Email: Miquel A. Pericàs - mapericas@iciq.es

* Corresponding author

Detailed experimental procedures for the preparation of the catalysts and chromatographic methods for the determination of the enantiomeric excess of the products

Table of contents

1. General methods S2
2. Synthesis of the immobilized catalysts S2
3. HPLC data for β-aminoxy alcohols S5
4. References S6

1. General methods

Unless otherwise stated, all commercial reagents were used as received. Merrifield resin (1% DVB, f = 0.53 mmol of Cl g^{-1} resin) was obtained from Novabiochem. Flash chromatography was carried out with 60 mesh silica gel and dry-packed columns. NMR spectra were registered in a Bruker Advance 400 Ultrashield spectrometer in $CDCl_3$ at room temperature, operating at 400.13 MHz (^{1}H) and 100.63 MHz (^{13}C{1H}). TMS was used as internal standard for ^{1}H NMR and $CDCl_3$ for ^{13}C NMR. IR spectra were recorded on a Bruker Tensor 27 / Diamond ATR FT-IR spectrometer and are reported in wavenumbers (cm^{-1}). Elemental analyses were performed on a LECO CHNS 932 microanalyzer at the Universidad Complutense de Madrid, Spain. High performance liquid chromatography (HPLC) was performed on Agilent Technologies chromatographs (Series 1100 and 1200), by using Chiralpak AD-H columns (with AD-H pre-columns). Racemic standard products were prepared with DL-proline as catalyst in order to establish HPLC conditions. The spectroscopic data of all β-aminoxy alcohols were identical to data reported in the literature [1-3].

2. Synthesis of the immobilized catalysts (1a,b)

Synthesis of *O-tert*-butyl-*N*,*N'*-diisopropylisourea [4]

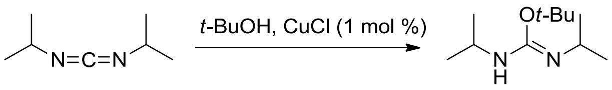

CuCl (259 mg, 3.63 mmol, 1 mol %) was added to a solution of *N*,*N'*-diisopropylcarbodiimide (56.8 mL, 45.8 g, 363 mmol) in dry *t*-BuOH (39.7 mL, 417 mmol). The reaction mixture was stirred for 14 h at rt. A ^{1}H NMR spectrum was recorded to check that the reaction was completed. After filtration most of the *t*-BuOH was removed from the filtrate under reduced pressure. The product (58.9 g, 294 mmol, 81%) was used directly in the next reaction without any further purification.

All spectroscopic data of the product were identical to those reported in the literature [4].

Synthesis of *tert*-Butyl (2*S*,4*R*)-*N*-Boc-4-hydroxyprolinate

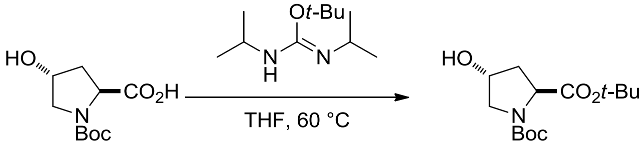

A solution of (2*S*,4*R*)-*N*-Boc-4-hydroxyproline (3.96 g, 17.1 mmol) in 60 mL of dry THF was treated with *O*-*tert*-butyl-*N,N'*-diisopropylisourea **7** (5.14 g, 25.7 mmol) at rt and then stirred for 2.5 h at 60 °C. Additional *O*-*tert*-butyl *N,N*'-diisopropylisourea **7** (3.13 g, 17.1 mmol) was added to the reaction mixture, and then stirring was continued overnight. Precipitated urea was filtered through a pad of Celite followed by washings with ether. The organic solution was concentrated under reduced pressure. Purification by flash column chromatography on silica gel (hexanes/EtOAc 8:2) afforded the title product as a colorless oil. (3.93 g, 80% yield).

All spectroscopic data of the product were in agreement with those reported in the literature [5].

Synthesis of *tert*-Butyl (2*S*,4*R*)-*N*-Boc-4-propargyloxyprolinate (2)

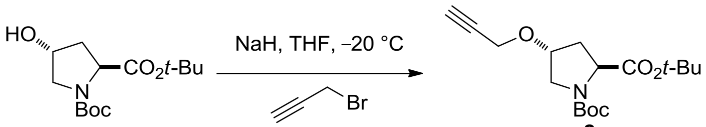

2

To a solution of *tert*-butyl (2*S*,4*R*)-*N*-Boc-4-hydroxyprolinate (1.00 g, 3.5 mmol) in anhydrous THF (50 mL) at −20 °C, sodium hydride (0.17 g NaH 60% in mineral oil, 4.2 mmol) was added. The so-obtained mixture was stirred under nitrogen atmosphere for 1 h at −20 °C, after which propargyl bromide (0.46 mL propargyl bromide 80% in toluene, 4.1 mmol) was added dropwise. The reaction mixture was allowed to slowly reach rt and stirred under nitrogen atmosphere for 16 h. The reaction was then quenched with methanol (5 mL) and subsequently with water (50 mL). After that, the mixture was extracted with ethyl acetate (3 x 50 mL), the organic layers were combined, treated with brine (25 mL) and dried over anhydrous Na_2SO_4. The crude product, obtained after filtration and evaporation of the solvents under reduced pressure, was purified by flash column chromatography on silica gel (hexanes/EtOAc 8:2) to afford 0.74 g (65% yield) of the title compound as a yellow oil.

All spectroscopic data of the product were in agreement with those reported in the literature [6].

Synthesis of azidomethyl resins (4a,b)

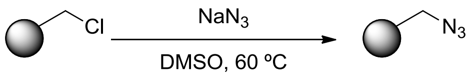

Resins **4a**,**b** were prepared by reported procedures [6]. A slightly cross-linked (1% 1,4-divinylbenzene) Merrifield resin with a functionalization of 0.53 mmol Cl g^{-1} and a home-made Merrifield resin (8% 1,4-divinylbenzene, 0.80–1.00 mmol Cl g^{-1}) were used as starting materials for **4a** and **4b**, respectively. In each case, the extent of the resin functionalization with azide groups was calculated by using the %N determined by elemental analysis.

4a IR (ATR): ν (cm^{-1}) = 2921, 2097, 1601, 1492, 1451. Elemental analysis (%) = N, 2.21; C, 88.84; H, 7.62. f = 0.53 mmol g^{-1}.

4b IR (ATR): ν (cm^{-1}) = 2920, 2093, 1600, 1491, 1451. Elemental analysis (%) = N, 3.71; C, 87.32; H, 7.48. f = 0.88 mmol g^{-1}.

Synthesis of the immobilized catalysts (1a,b)

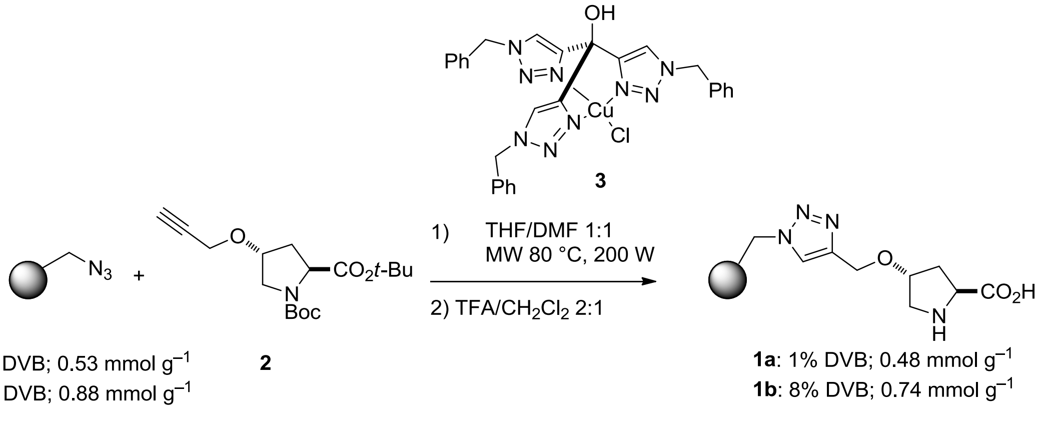

<u>1) Cycloaddition of *tert*-butyl (2*S*,4*R*)-*N*-Boc-4-propargyloxyprolinate with **4a**,**b**</u>

600 mg of resin **4a**,**b** together with 1.2 equiv of proline derivative **2** were placed in a microwave reactor tube along with 4 mL of DMF and 4 mL of THF. Then, tris(1-benzyl-1*H*-1,2,3-triazol-4-yl)methanol·CuCl catalyst **3** [7] (2 mol %) was added. The reaction mixture was heated at 80 °C for 60–150 min under microwave irradiation at 200 W, without stirring. After the reaction was

complete (as determined by the disappearance of the absorption band at ca. 2095 cm^{-1} in the IR spectrum), the resin was filtered and sequentially washed with water (100 mL), water/methanol 1:1 (100 mL), methanol (100 mL), THF/methanol 1:1 (100 mL), THF (100 mL) and dichloromethane (100 mL). The resulting resin was dried in vacuo at 40 °C for 24 h, before being submitted to the next step.

2) General procedure for *N*-Boc and *tert*-butylester deprotection

The resin resulting from the previous step was swollen with 5 mL of CH_2Cl_2. After 10 min, 10 mL of trifluoroacetic acid was added and the evolution of the deprotection was followed by ATR-FTIR. The reaction mixture was filtered when the IR-signal of *t*-Bu and carbonyl groups had completely disappeared. After filtration, polystyrene-supported proline **1a**,**b** was sequentially washed with THF (with 2% of Et_3N, 100 mL), water (100 mL), THF (100 mL), THF/MeOH 1:1 (100 mL), MeOH (100 mL) and THF (100 mL). The solid was dried in vacuo for 24 h at 40 °C.

1a IR (ATR): ν (cm^{-1}) = 2919, 1596, 1491, 1447. Elemental analysis (%) = N, 2.68; C, 85.28; H, 7.32. f = 0.48 mmol g^{-1}.

1b IR (ATR): ν (cm^{-1}) = 2920, 1600, 1492, 1451. Elemental analysis (%) = N, 4.13; C, 80.40; H, 7.17. f = 0.74 mmol g^{-1}.

3. HPLC data for β-aminoxy alcohols

(*R*)-2-(*N*-Phenyl-aminoxy)propan-1-ol [1]

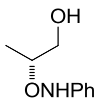

Daicel CHIRALCEL AD-H column, hexane/2-propanol 95:5, 1 L/min, λ = 240 nm, t_R (minor): 22.6 min, t_R (major): 26.4 min.

(*R*)-3-Methyl-2-(*N*-phenyl-aminoxy)butan-1-ol [1]

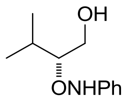

Daicel CHIRALCEL AD-H column, hexane/2-propanol 95:5, 1 L/min, λ = 240 nm, t_R (minor): 17.3 min, t_R (major): 20.3 min.

(*R*)-2-(*N*-Phenyl-aminoxy)pentan-1-ol [1]

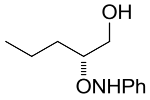

Daicel CHIRALCEL AD-H column, hexane/2-propanol 95:5, 1 L/min, λ = 240 nm, t_R (minor): 20.1 min, t_R (major): 23.9 min.

(*R*)-2-(*N*-Phenyl-aminoxy)undec-10-en-1-ol [2]

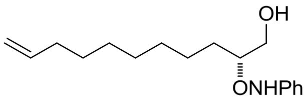

Daicel CHIRALCEL AD-H column, hexane/2-propanol 95:5, 1 L/min, λ = 240 nm, t_R (minor): 15.0 min, t_R (major): 22.3 min.

(*R*)-2-(*N*-Phenyl-aminoxy)butan-1-ol [3]

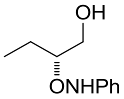

Daicel CHIRALCEL AD-H column, hexane/2-propanol 95:5, 1 L/min, λ = 240 nm, t_R (minor): 20.4 min, t_R (major): 23.6 min.

4. References

1. Zhong, G. *Angew. Chem. Int. Ed.* **2003**, *42*, 4247–4250.
2. Font, D.; Bastero, A.; Sayalero, S.; Jimeno, C.; Pericàs, M. A. *Org. Lett.* **2007**, *9*, 1943–1946.
3. Hayashi, Y.; Yamaguchi, J.; Sumiya, T.; Hibino, K.; Shoji, M. *J. Org. Chem.* **2004**, *69*, 5966–5973.
4. (a) Brasche, G.; Grube, A.; Böhnke, N.; Stadler, C.; Tietze, L. F. *Chem. Eur. J*. **2007**, *13*, 8543. (b) West, K. R.; Bake, K. D.; Otto, S. *Org .Lett*. **2005**, *7*, 2615. (c) Mathias, L. J . *Synthesis* **1979**, *8*, 561.
5. Helaja, J.; Kumpulainen, E. T. T.; Koivisto, J.; Mansikkamäki, H.; Rissanen, K. ; Koskinen, A. M. P. *J. Org. Chem.* **2005**, *70*, 6447.
6. Font, D.; Jimeno, C.; Pericàs, M. A. *Org. Lett.* **2006**, *8*, 4653–4655.
7. Özçubukçu, S.; Ozkal, E.; Jimeno, C.; Pericàs, M. A. *Org. Lett.* **2009**, *11*, 4680–4683.
